# Supplementary material for: Automated Radiosynthesis, Preliminary In Vitro/In Vivo Characterization of OncoFAP-Based Radiopharmaceuticals for Cancer Imaging and Therapy
Source: Pharmaceuticals (Basel). 2022 Aug 2;15(8):958. doi: 10.3390/ph15080958 (PMC9416253; doi:10.3390/ph15080958)
Supplement: Supplementary file 1 [file pharmaceuticals-15-00958-s001.zip › pharmaceuticals-1807687-supplementary.pdf]

Automated radiolabeling procedures for the preparation of OncoFAP-based radiopharmaceuticals for cancer imaging and therapy

Francesco Bartoli[1], Philip Elsinga[2], Luiza Reali Nazario[2], Aureliano Zana[3], Andrea Galbiati[3], Jacopo Millul[3], Francesca Migliorini[3], Samuele Cazzamalli[3], Dario Neri[4,5], Riemer H.J.A. Slart[2,6] and Paola Anna Erba[1,2]

[1]Nuclear Medicine, Department of Translational Research and Advanced Technologies in Medicine and Surgery, University of Pisa and Azienda Ospedaliero Universitaria Pisana, 56126 Pisa, Italy; francesco.bartoli@med.unipi.it (F.B.); paola.erba@unipi.it (P.A.E.)
[2]University of Groningen, University Medical Center Groningen, Medical Imaging Center, Groningen, The Netherlands
[3]Philochem AG, R&D department, Libernstrasse 3, CH-8112 Otelfingen (ZH), Switzerland
[4]Swiss Federal Institute of Technology, Department of Chemistry and Applied Biosciences, CH-8093 Zurich, Switzerland
[5]Philogen S.p.A., 53100 Siena, Italy
[6]Biomedical Photonic Imaging Group, Faculty of Science and Technology, University of Twente, Enschede, The Netherlands.

Chemical synthesis

All reagents and solvents were purchased from Sigma Aldrich, VWR, CheMatech, and Combi-Blocks and used as supplied.

High Pressure Liquid Chromatography/Mass-Spectrometry (HPLC-MS) spectra presented were recorded on an Agilent 6100 Series Single Quadrupole MS system combined with an Agilent 1200 Series LC, using an InfinityLab Poroshell 120 (EC-C18 column, 4.6 × 50 mm, particle size: 2.7 µm, pore size: 120 Å) at a flow rate of 0.8 mL/ min.

Time (min)	Water + 0.1% HCOOH	ACN + 0.1% HCOOH	Flow (mL/min)
0.00	90.0 %	10 %	0.800
0.50	90.0 %	10 %	0.800
3.00	0.0 %	100 %	0.800
3.50	0.0 %	100%	0.800

Reversed-phase high-pressure liquid chromatography (RP-HPLC) were performed on an Agilent 1200 Series RP-HPLC with PDA UV detector, using a Synergi MAX-RP (C18 column, 10 × 250 mm, particle size: 10 µm, pore size: 80 Å) at a flow rate of 5 mL/min with linear gradients of solvents A and B (A = Millipore water with 0.1% TFA, B = ACN with 0.1% TFA).

General synthetic scheme of NOTA-OncoFAP

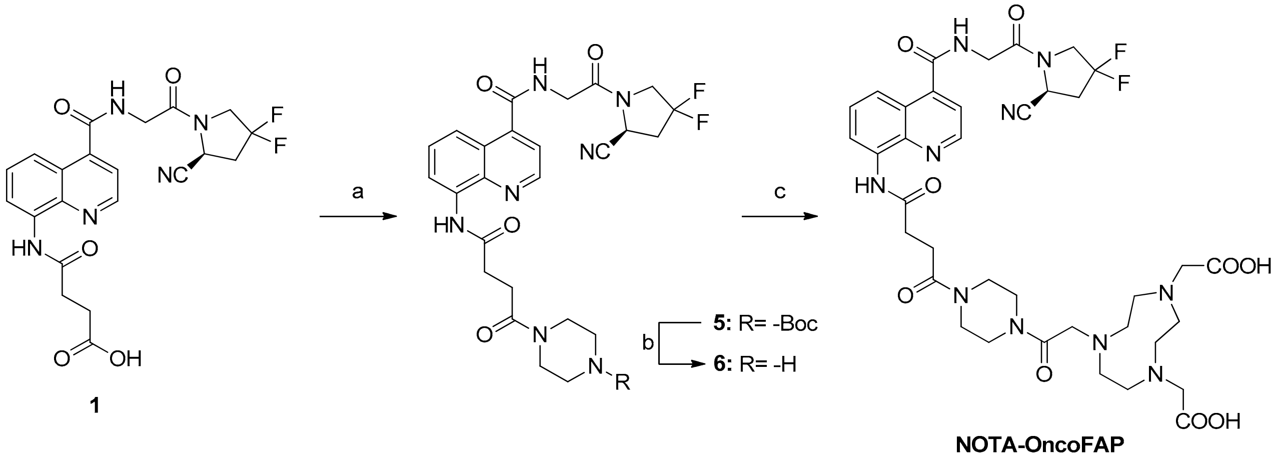

a) *N*-Boc-piperazine, HATU, DIPEA, DMF, r.t., 30 min; b) TFA/DCM 30% v/v, r.t., 3h; c) NOTA-NHS, DIPEA, DMF, r.t., 1h.

*Synthesis of tert-butyl (S)-4-(4-((4-((2-(2-cyano-4,4-difluoropyrrolidin-1-yl)-2-oxoethyl)carbamoyl)quinolin-8-yl)amino)-4-oxobutanoyl)piperazine-1-carboxylate (**5**)*

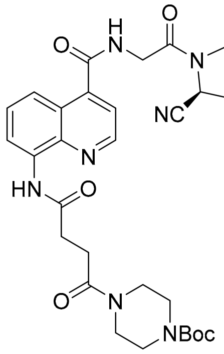

(*S*)-4-((4-((2-(2-cyano-4,4-difluoropyrrolidin-1-yl)-2-oxoethyl)carbamoyl)quinolin-8-yl)amino)-4-oxobutanoic acid (compound **1**) (50 mg, 0.11 mmol, 1 eq), *N*-Boc-piperazine (24 mg, 0.13 mmol, 1.2 eq) and HATU (49 mg, 0.13 mmol, 1.2 eq) were dissolved in DMF (1 mL). DIPEA (0.08 mL, 0.44 mmol, 4 eq) was added dropwise and the mixture was stirred for 30 min at room temperature. The mixture was purified via RP flash chromatography (Büchi Sepacore 40 µm irregular column) using a gradient of 2:98 to 100:0 ACN + 0.1% HCOOH/water + 0.1% HCOOH in 40 min. The desired fractions were collected and lyophilized to afford a white solid. (40 mg, 58%)

MS (ESI+) m/z 627.9 [M+H]

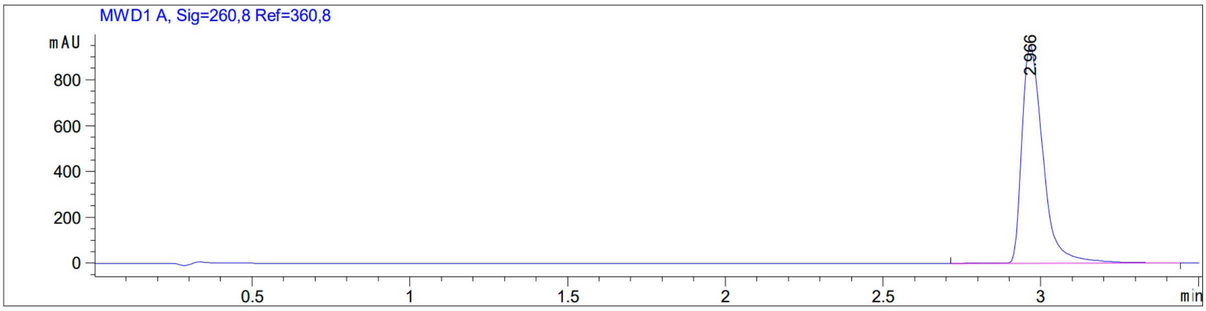

*Synthesis of (S)-N-(2-(2-cyano-4,4-difluoropyrrolidin-1-yl)-2-oxoethyl)-8-(4-oxo-4-(piperazin-1-yl)butanamido)quinoline-4-carboxamide (**6**)*

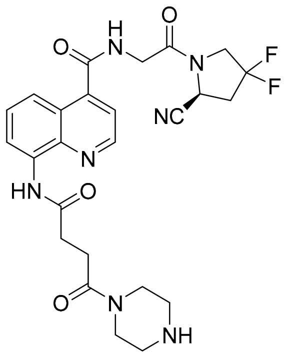

Compound **5** (40 mg, 0,06 mmol, 1 eq.) was dissolved in DCM (1 mL) and TFA (0.5 mL) was added dropwise. The mixture was stirred at room temperature for 3h then solvent was evaporated, and the crude was purified via RP flash chromatography (Büchi Sepacore 40 µm irregular column) using a gradient of 2:98 to 100:0 ACN + 0.1% HCOOH/water + 0.1% HCOOH in 40 min. The desired fractions were collected and lyophilized to afford a colorless oil. (17 mg, 51%)

MS (ESI+) m/z 527.9 $[M+H]^+$

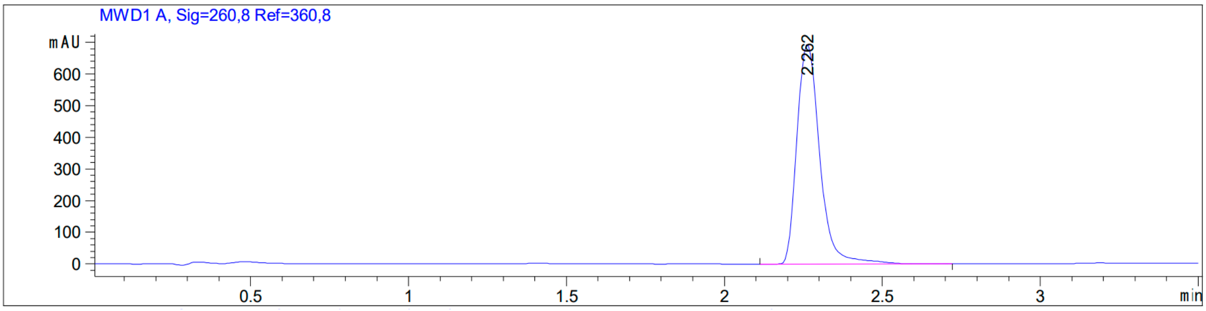

*Synthesis of (S)-2,2'-(7-(2-(4-(4-((4-((2-(2-cyano-4,4-difluoropyrrolidin-1-yl)-2-oxoethyl)carbamoyl)quinolin-8-yl)amino)-4-oxobutanoyl)piperazin-1-yl)-2-oxoethyl)-1,4,7-triazonane-1,4-diyl)diacetic acid, **NOTA-OncoFAP** (**4**)*

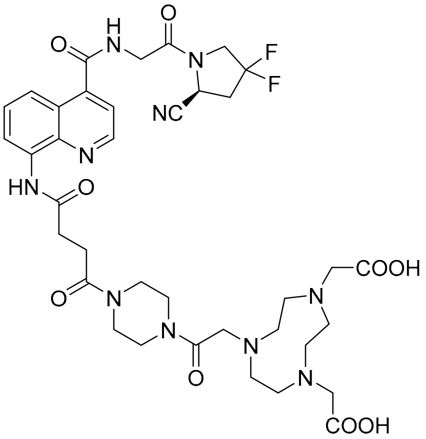

Compound **6** (8 mg, 0.02 mmol, 1 eq) and NOTA-NHS (19 mg, 0.04 mmol, 2 eq) were dissolved in DMF (0.2 mL). DIPEA (0.01 mL, 0.08 mmol, 4 eq) was added to the mixture and stirred for 1h at room temperature. The mixture was purified via RP-HPLC (90:10 to 0:100 water + 0.1% TFA/ACN + 0.1% TFA in 12 min). The desired fractions were collected and lyophilized to afford a white solid. (4 mg, 25%)

MS (ESI+) m/z 812.7 $[M+H]^+$

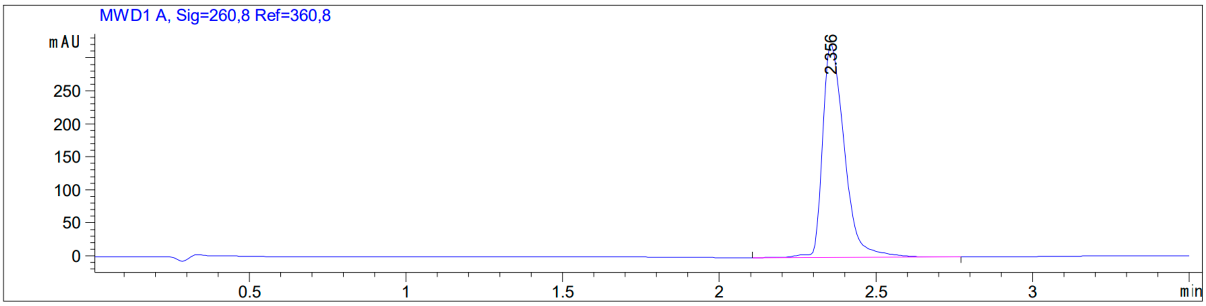

General scheme and synthesis of OncoFAP-NODAGA (3)

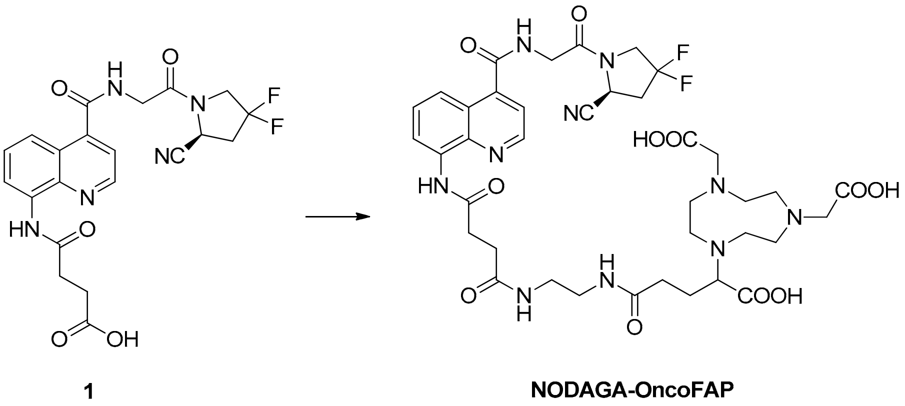

To a solution of compound **1** (50 mg, 0.11 mmol, 1 eq) in dry DMSO (1 mL) were added *N*-hydroxy succinimide (19 mg, 0.16 mmol, 1.5 eq), HATU (61 mg, 0.16 mmol, 1.5 eq) and DIPEA (57 µL, 0.44 mmol, 4 eq), the mixture at room temperature. After 30 min NODA-GA-NH2 (46 mg, 0.22 mmol, 2 eq) and water (0.3 mL) were added, and the mixture was stirred for 2h. The reaction mixture was directly purified via RP-HPLC (90:10 to 0:100 water + 0.1% TFA/ACN + 0.1% TFA in 12 min). The desired fractions were collected and lyophilized to afford a white solid. (30 mg, 32%)

MS (ESI+) m/z 859.4 $[M+H]^+$

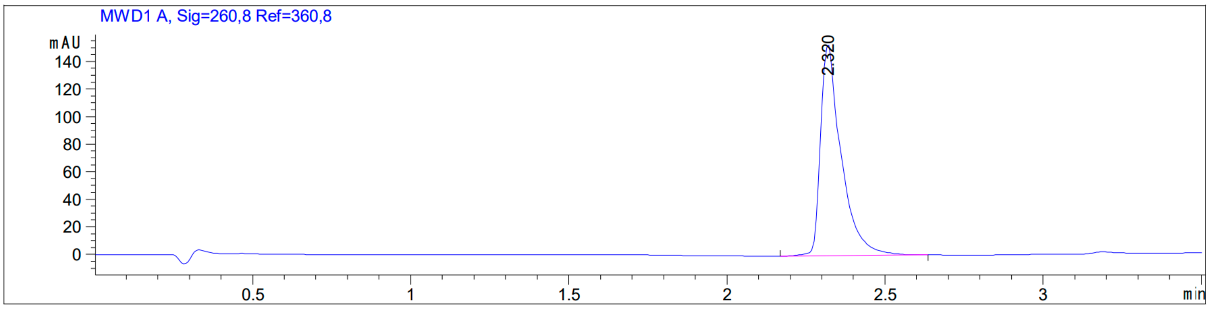

NODAGA-OncoFAP labeling with ^{177}Lu and characterization

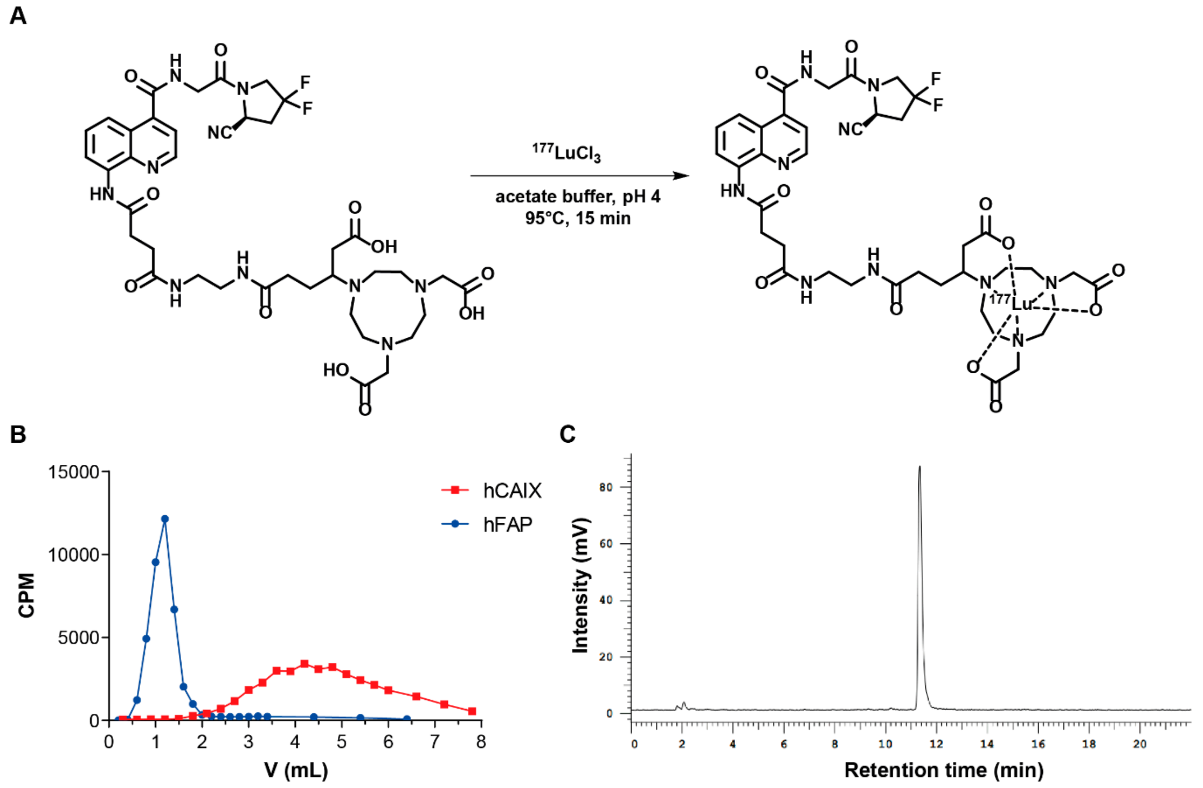

Figure S1. (*A*) Radiolabeling reaction scheme of OncoFAP-NODAGA with ^{177}Lu in acetate buffer (pH=4) at 95°C for 15 min. (*B*) Coelution experiment of [^{177}Lu]Lu-NODAGA-OncoFAP- with hFAP and hCAIX on PD-10 size exclusion chromatography columns. (*C*) Radio-HPLC analysis performed after labeling reaction.

NODAGA-OncoFAP can be efficiently labeled with ^{177}Lu by heating at 95°C in acetate buffer (pH=4) for 15 min. [**Figure S1A**] After labeling with 1 MBq activity the Radio-HPLC trace shows complete incorporation of ^{177}Lu and a single peak profile (no product degradation observed) [**Figure S1B**]. The coelution experiment confirms the strong affinity of [^{177}Lu]Lu-NODAGA-OncoFAP for hFAP, while no significant interaction with hCAIX, as a non-target protein, has been observed. [**Figure S1C**]

Radiosynthesis of [^{68}Ga]Ga-OncoFAP

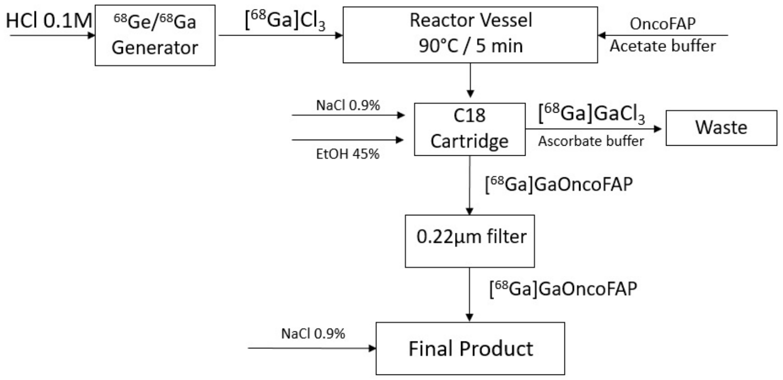

Figure S2. flow chart for radiosynthesis of [^{68}Ga]Ga-OncoFAP

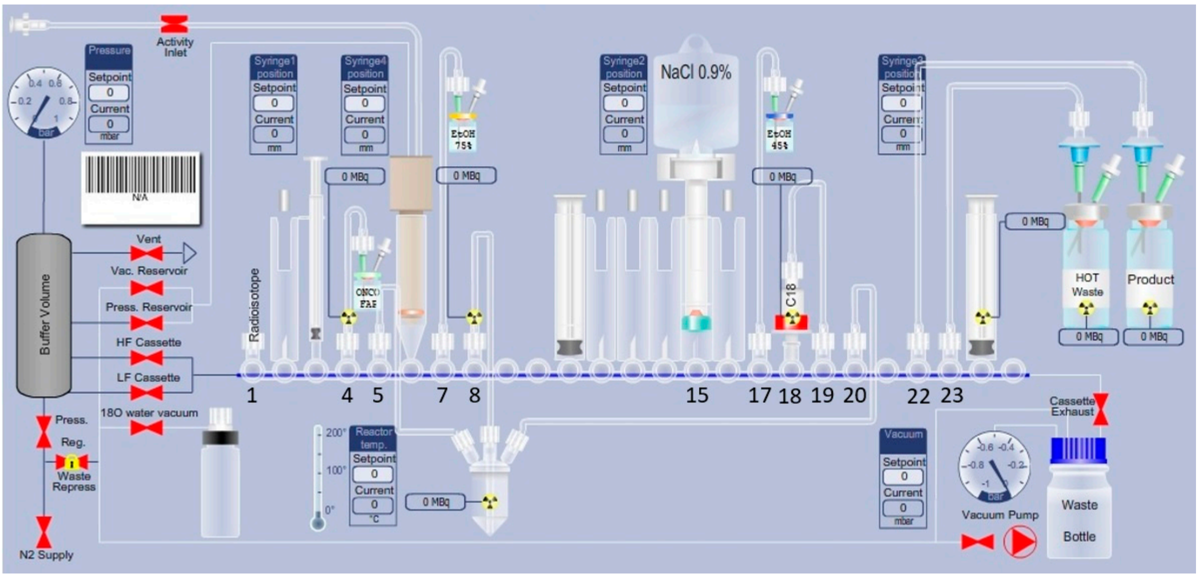

Figure S3. Layout of the cassette for radiosynthesis of [^{68}Ga]Ga-OncoFAP derivatives. Position: 1) Generator; 4) Vial containing precursor dissolved in 0.7 M sodium acetate solution (650 µL); 5, 8, 20) Reactor; 7) Awaking solution (75% EtOH); 15) 0.9% NaCl solution; 17) Eluent solution (45% EtOH); 18, 19) C18 cartridge; 22) Product vial; 23) Hot waste.

Radiosynthesis of [^{18}F]AlF-NOTA-OncoFAP

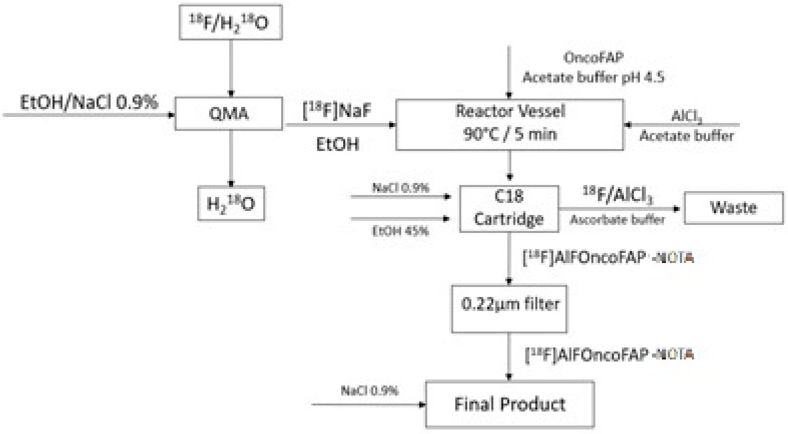

Figure S4. Flow chart for radiosynthesis of [^{18}F]AlF-OncoFAP

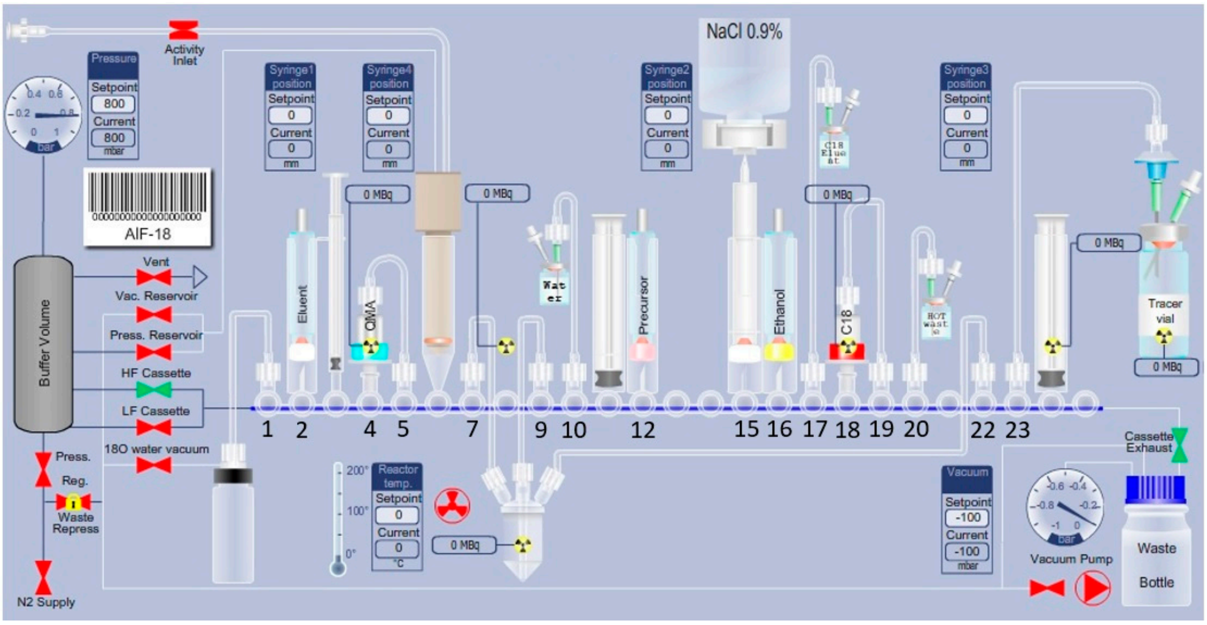

Figure S5. Layout of the cassette for radiosynthesis of [^{18}F]AlF-OncoFAP-; 1) Recovery $H_2{}^{18}O$; 2) QMA Eluent; 4,5) QMA cartridge; 6) Conical vial connected to vial with ^{18}F; 7, 9, 22) Reactor; 10) OncoFAP-NOTA solution dissolved in acetate buffer pH 4.1; 15) 0.9% NaCl solution; 16) Awaking solution (75% EtOH); 17) Eluent solution (45% EtOH); 18-19) C18 cartridge; 20) Hot Waste; 22) Product Vial.

HPLC purification

Fluorine-18 radiolabelling of NODAGA-OncoFAP was achieved as follow: [^{18}F]Fluorine (647 MBq) was trapped in the cartridge (45mg $PS\text{-}HCO_3^-$) and washed with 3 mL metal-free H_2O and dried with 7 mL air. The cartridge was then eluted with 100 µL of 0.9% NaCl solution directly in the $AlCl_3$ solution (2 mM; pH 4.7; 10 µL) and reacted for 5 min. 10 µL of OncoFAP-NODAGA in sodium acetate (0.5 M, pH 4, 50 µg) and 200 µL of ACN was added to the solution and the resulting reaction mixture was heated at 100°C for 20 min. After the labelling, the

mixture was injected on HPLC (Synergy 4 µm Hydro-RP 80Å; 20/80 ACN/water TFA 0.1% gradient 30min). The product peak was collected at 5.8 min and diluted in 60 µL of water. The solution passed through an Oasis® HLB 1 cc/30 mg and the product was eluted with 400 µL ethanol and reconstituted with 0.9% NaCl solution (5 mL). The RCY (decay corrected) of this reaction is 3% and the RCP >95%.

Radiosynthesis of [^{177}Lu]Lu-DOTAGA-OncoFAP

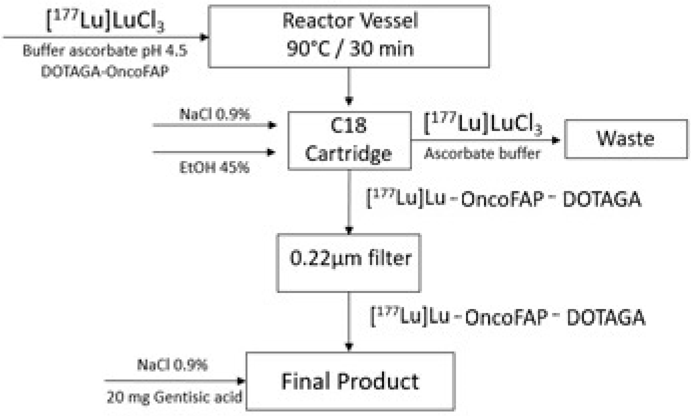

Figure S6. Flow chart for radiosynthesis of [^{177}Lu]Lu-DOTAGA-OncoFAP

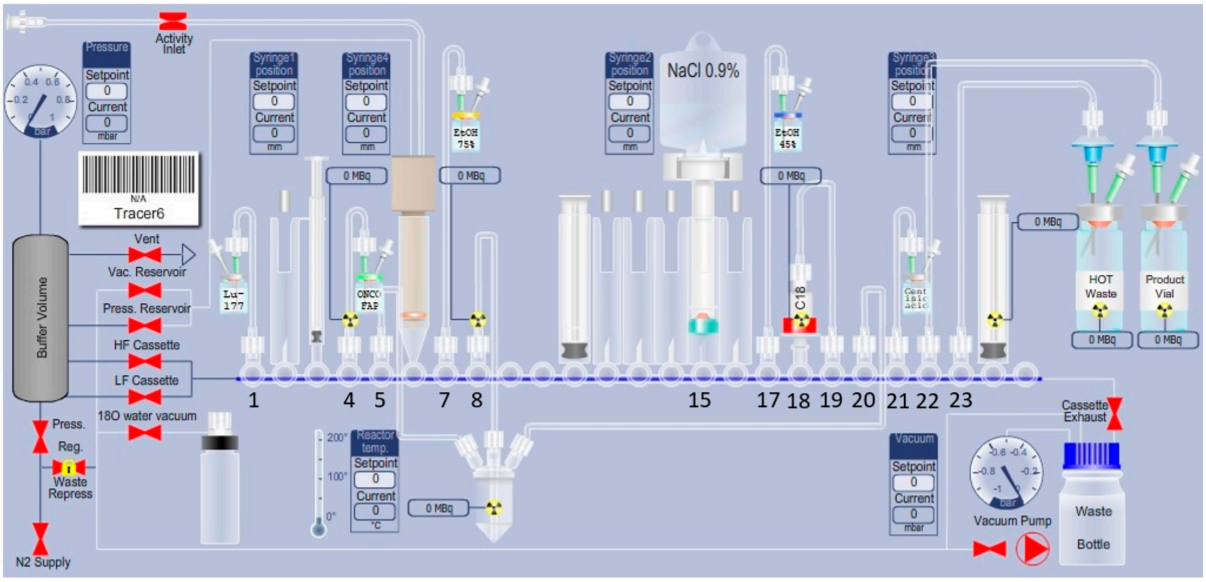

Figure S7. Layout of the cassette for radiosynthesis of [^{177}Lu]Lu-DOTAGA-OncoFAP; Position: 1). Vial of ^{177}Lu in acetate buffer pH 4.5; 4) DOTAGA-OncoFAP in acetate buffer pH 4.5; 5, 8, 20) Reactor; 7) Awaking solution (75% EtOH); 15) 0.9% NaCl solution; 16) Awaking solution (75% EtOH); 17) Eluent solution (45% EtOH); 18,19) C18 cartridge; 21) Gentisic Acid dissolved in 0.9% NaCl solution; 22) Product vial; 23) Hot Waste.

Radio-HPLC analysis before and after labelling reaction of [^{68}Ga]Ga-DOTAGA-OncoFAP

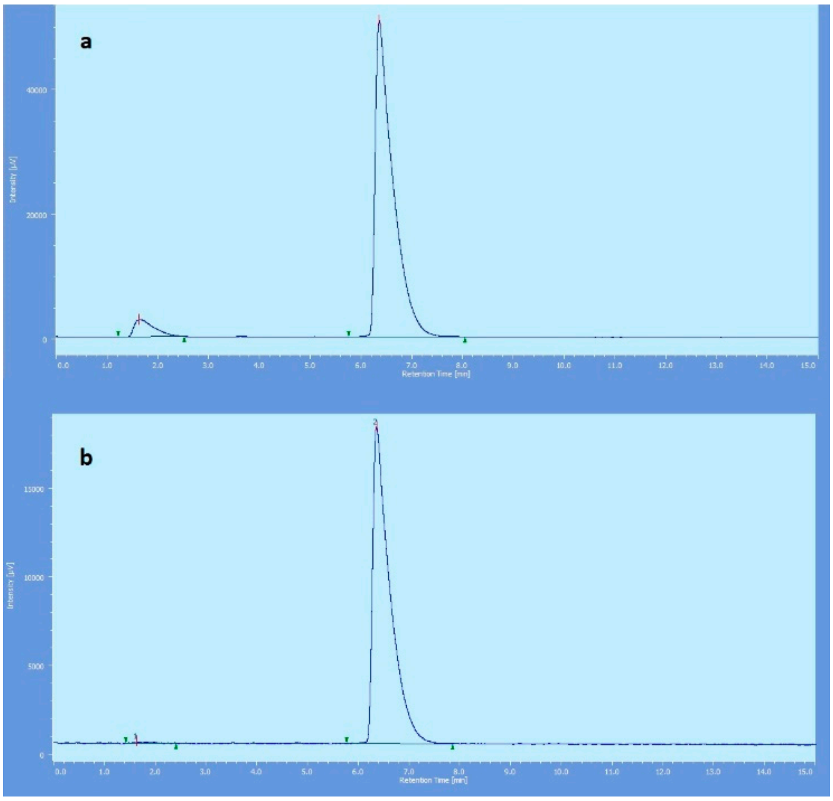

Figure S8. Radio-HPLC trace performed after radiosynthesis of [^{68}Ga]Ga-DOTAGA-OncoFAP (*A*) before purification with C18 cartridge and (*B*) after purification with C18.

Radio-HPLC analysis after labelling reaction of [^{177}Lu]Lu-DOTAGA-OncoFAP

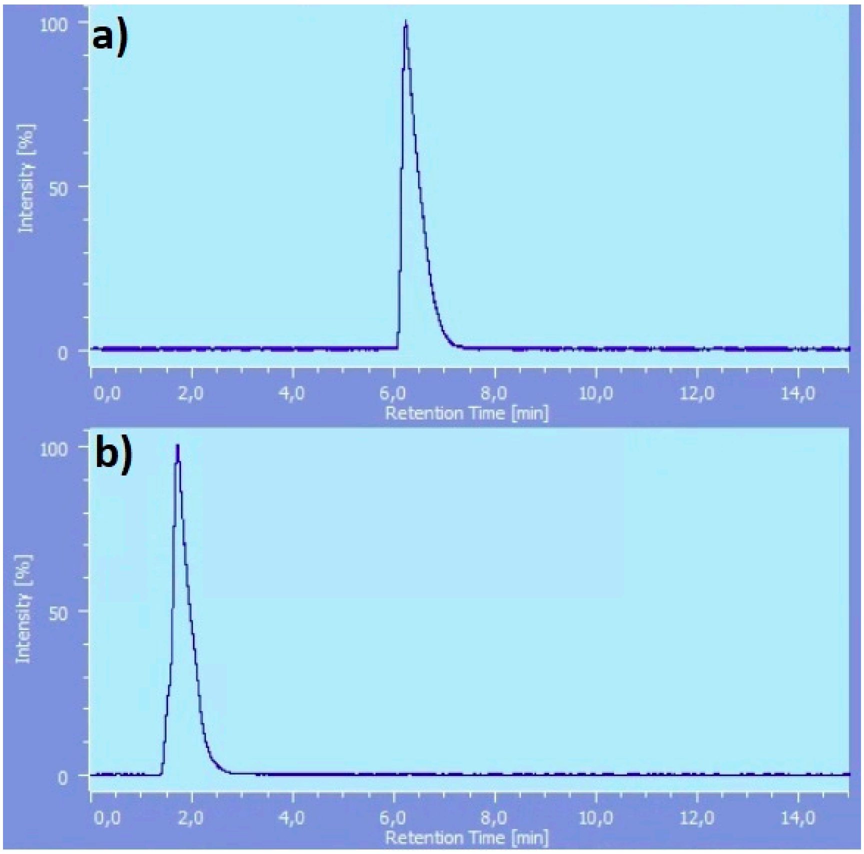

Figure S9. Radio-HPLC trace performed after radiosynthesis of [^{177}Lu]Lu-DOTAGA-OncoFAP (*A*) product (*B*) "Hot" waste of the synthesis

Co-elution experiments of [^{68}Ga]Ga-NOTA-OncoFAP; [^{68}Ga]Ga-NODAGA-OncoFAP, [^{177}Lu]Lu-DOTAGA-OncoFAP and [^{18}F]AlF-NOTA-OncoFAP with hFAP

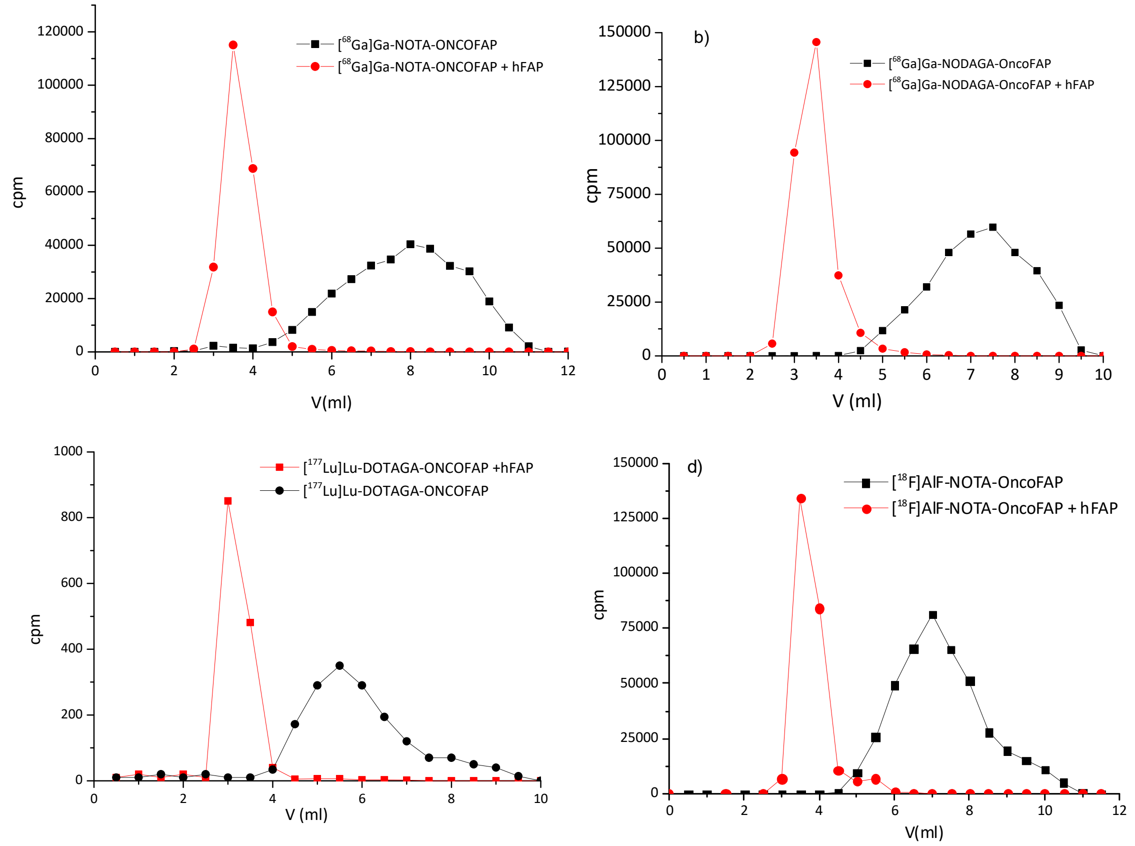

Figure S10. Co-elution experiment of radiolabelled preparations of (*A*) [^{68}Ga]Ga-NOTA-OncoFAP; (*B*) [^{68}Ga]Ga-NODAGA-OncoFAP (*C*) [^{177}Lu]Lu-DOTAGA-OncoFAP (*D*) [^{18}F]AlF-NOTA-OncoFAP with hFAP and without hFAP on PD-10 size exclusion chromatography columns.

Radiolabeling Simple kit

In all synthesis we used a reaction time of 10 minutes, a reaction temperature of 95°C and we have obtained a final volume of 4.5ml.

**Radiosynthesis [^{68}Ga]Ga-NODAGA-OncoFAP Formate buffer (Simple Preparation)**			
	Batch 1	Batch 2	Batch 3
**NODAGA-OncoFAP (µg)**	10	20	40
**Starting activity (GBq)**	1.11±0.3	1.13±0.3	1.05±0.3
**- Free gallium-68 content**	6.2±0.3%	3.0±0.3%	1.3±0.3%
**- Colloidal ^{68}Ga content**	1.3±0.3%	1.7±0.3%	1.5±0.3%
**- Radiochemical purity**	92.5±0.3%	95.3±0.3%	97.2±0.3%

Table S1. Different conditions used for the synthesis [^{68}Ga]Ga-NODAGA-OncoFAP

Radiosynthesis [^{68}Ga]Ga-NODAGA-OncoFAP<br>Acetate buffer (Simple Preparation)			
	Batch 1	Batch 2	Batch 3
**NODAGA-OncoFAP (µg)**	10	20	40
**Starting activity (GBq)**	1.15±0.3	0.98±0.3	1.12±0.3
**- Free gallium-68 content**	14.2±0.3%	10.3±0.3%	6.3±0.3%
**- Colloidal ^{68}Ga content**	7.5±0.3%	7.4±0.3%	6.5±0.3%
**- Radiochemical purity**	78.3±0.3%	82.3±0.3%	87.2±0.3%

Table S2. Different conditions used for the synthesis [^{68}Ga]Ga-OncoFAP-NODAGA

Radiosynthesis [^{68}Ga]Ga-DOTAGA-OncoFAP<br>Formate buffer (Simple Preparation)			
	Batch 1	Batch 2	Batch 3
**DOTAGA-OncoFAP**	10	20	40
**Starting activity (GBq)**	1.04±0.3	0.97±0.3	0.93±0.3
**- Free gallium-68 content**	12.9±0.3%	4.2±0.3%	3.3±0.3%
**- Colloidal ^{68}Ga content**	1.9±0.3%	1.7±0.3%	1.7±0.3%
**- Radiochemical purity**	85.2±0.3%	94.1±0.3%	95.0±0.3%

Table S3. Different conditions used for the synthesis [^{68}Ga]Ga-OncoFAP-NODAGA

Radiosynthesis [^{68}Ga]Ga-DOTAGA-OncoFAP<br>Acetate buffer (Simple Preparation)			
	Batch 1	Batch 2	Batch 3
**DOTAGA-OncoFAP**	10	20	40
**Starting activity (GBq)**	1.08±0.3	1.02±0.3	0.95±0.3
**- Free gallium-68 content**	14.7±0.3%	9.8%	6.6%
**- Colloidal ^{68}Ga content**	9.0%	8.7%	7.1%
**- Radiochemical purity**	76.3%	81.5%	86.3%

Table S4. Different conditions used for the synthesis [^{68}Ga]Ga-OncoFAP-NODAGA

Assessment of *in vitro* lipophilicity

Product	LogP	LogD$_{7.4}$
**[^{68}Ga]Ga-NOTA-OncoFAP**	-2.38± 0.11	-2.19± 0.11
**[^{68}Ga]Ga-NODAGA-OncoFAP**	-2.68± 0.14	-2.48± 0.09
**[^{68}Ga]Ga-DOTAGA-OncoFAP**	-2.57± 0.15	-2.39 ± 0.12
**[^{18}F]AlF-NOTA-OncoFAP**	-2.15± 0.18	-2.01± 0.25
**[^{18}F]AlF-NODAGA-OncoFAP**	-3.02± 015	-2.88±0.16
**[^{177}Lu]Lu-DOTAGA-OncoFAP**	-2.01± 0.08	-1.85± 0.05

Table S5. LogP and LogD$_{7.4}$
